# Patient Participation in Patient Safety Practices Scale: Development and Psychometric Evaluation of a Scale

**DOI:** 10.3390/healthcare13121387

**Published:** 2025-06-11

**Authors:** Meltem Dursun Engin, Şeyda Seren İntepeler

**Affiliations:** 1 Nursing Management Department, Ege University Faculty of Nursing, 35100 Izmir, Türkiye; 2 Nursing Management Program, Institute of Health Sciences, Dokuz Eylül University, 35340 Izmir, Türkiye; 3 Nursing Management Department, Dokuz Eylül University Faculty of Nursing, 35340 Izmir, Türkiye; seyda.seren@deu.edu.tr

**Keywords:** patient safety, patient participation, scale development, infection, patient fall

## Abstract

**Introduction:** Patient participation is a critical element in enhancing patient safety. Involving patients in safety practices improves communication, reduces errors, and optimizes treatment outcomes. However, there is no standardized instrument that measures patient participation in safety practices. **Methods:** This study was designed as a scale development and psychometric validation study to create the Patient Participation in Patient Safety Practices Scale (PPPSPS). The methodological research was conducted with 424 inpatients in a Turkish public hospital between June 2021 and February 2022. The scale development process included item generation, expert review, a pilot study, and statistical validation. Content validity was assessed using Lawshe’s content validity ratio (CVR). Structural validity was tested through exploratory factor analysis (EFA) and confirmatory factor analysis (CFA). Internal consistency reliability was evaluated using Cronbach’s alpha and item–total correlations. **Results:** The final version of the scale included 32 items under 4 subscales (general, infection, falls, and drugs). Cronbach’s alpha coefficient was 0.90 for the whole scale, 0.90 for the general subscale (11 items), 0.90 for the infection subscale (10 items), 0.81 for the fall subscale (6 items), and 0.80 for the drug subscale (5 items). EFA revealed four factors explaining 70.61% of the total variance. CFA confirmed a good model fit: χ² (457) = 1053.15; *p* < 0.001; χ²/df = 2.3; GFI = 0.930; AGFI = 0.920; CFI = 1.000; TLI = 0.981; RMSEA = 0.079; SRMR = 0.079. Cronbach’s alpha was 0.922 for the total scale and ranged between 0.799 and 0.932 for the subscales. **Conclusions:** The Patient Participation in Patient Safety Practices Scale is a valid and reliable tool for assessing patient participation in safety practices. It is recommended for use in clinical settings and further testing in different patient populations and healthcare systems.

## 1. Introduction

Patient safety refers to a set of organized activities in healthcare that aim to reduce the risk of preventable harm and medical errors while promoting systems and cultures that mitigate adverse events when they occur [1]. Ensuring patient safety is a shared responsibility among healthcare professionals, requiring effective teamwork, performance monitoring, and ongoing training [2]. Although healthcare professionals have traditionally been viewed as the primary agents in safety efforts, patients and their families are increasingly recognized as essential members of the care team [3,4].

Patients who are actively engaged in their care can contribute significantly to safety outcomes, such as reducing adverse events, preventing infections, and improving clinical decision-making [5,6]. The WHO [1,7] and various national bodies have emphasized the importance of involving patients in decisions related to their care to improve both the quality and safety of healthcare delivery. Patient participation in safety includes behaviors like asking questions, verifying medications, ensuring hand hygiene, and collaborating in fall prevention strategies [8,9].

This participation is grounded in a shift from “care for the patient” to “care with the patient”, empowering individuals to take a more active role in managing their health [10,11]. Key aspects include mutual communication with healthcare professionals, being informed, having control in decision-making, and engaging in preventive actions [12,13]. The literature highlights that increased participation not only improves satisfaction and health outcomes but also reduces healthcare costs, especially in managing chronic illnesses [14].

Although several instruments have been developed to measure patient participation—such as the Patient Activation Measure (PAM), the Patient Health Engagement (PHE) Scale, and tools specific to chronic disease self-management [15,16,17]—these instruments predominantly focus on general health behaviors, motivation, or empowerment in treatment decisions. They do not capture safety-specific participation behaviors that occur during hospitalization, such as medication checking, fall prevention, or infection control practices. Recent reviews have confirmed the lack of validated tools that directly assess patient participation in safety behaviors within clinical settings [18,19].

To address this critical gap, the present study introduces the Patient Participation in Patient Safety Practices Scale (PPPSPS), developed to measure patient participation specifically in safety-related activities across four domains: general safety, infection prevention, fall prevention, and medication safety. This scale offers a unique contribution by being, to the best of our knowledge, the first instrument tailored to comprehensively assess patient participation in safety practices in the Turkish and international literature.

The PPPSPS is highly relevant within the Turkish healthcare context, where national quality standards now emphasize patient-centered safety indicators [20]. Moreover, the core domains it evaluates—communication, shared responsibility, and active safety behaviors—align with global patient safety priorities, making the tool suitable for cultural adaptation and cross-national use [1,5]. The PPPSPS is expected to inform clinical practice, support safety-oriented policies, and guide quality improvement efforts by offering measurable insights into the patient’s role in enhancing healthcare safety. The PPPSPS can be implemented in various clinical settings, particularly in inpatient medical and surgical units, where patient involvement in safety practices is vital for preventing adverse events. It can be utilized by nurses, patient safety officers, and quality improvement teams to assess and monitor the level of patient participation in safety practices. The scale may also be incorporated into routine patient assessments, safety rounds, or discharge planning protocols to identify areas where additional education or support is needed. Furthermore, the tool can inform the design of patient engagement interventions, staff training programs, and institutional safety policies aimed at fostering a culture of patient-centered safety.

### The Aim of the Research

Research was conducted to develop the “Patient Participation in Patient Safety Practices Scale” (PPPSPS) to determine the level of patient participation in patient safety practices and examine its psychometric properties.

## 2. Materials and Methods

### 2.1. Research Design

The research was carried out via methodological design.

### 2.2. Settings

It was conducted at a training and research hospital affiliated with the Ministry of Health with a capacity of 517 beds and 550 nurses. The practices in the institution were being carried out within the scope of the Ministry of Health Quality Standards, including patient safety; however, no practices related to patient participation were included.

### 2.3. Research Sample

The population of the study consisted of inpatients admitted to a training and research hospital between June 2021 and February 2022. The sample of the study consisted of patients who were aged 18 years and older, stayed at the clinic for at least two nights in the internal and surgical departments of the hospital between these dates, and volunteered to participate in the study. The sample size, which is a general rule in scale validity and reliability studies, should be 5 or even 10 times the minimum number of variables, which was achieved in the present study [21,22,23]. The number of items on the scale was 32, the minimum number of samples with 5 items was determined to be 160, and the maximum number of samples was 320.

### 2.4. Sample Size Justification

The sample size was determined based on widely accepted methodological guidelines for scale development, which recommend a participant-to-item ratio ranging from 5:1 to 10:1 [24,25]. Considering the initial scale included 32 items, a sample size between 160 and 320 was considered sufficient. In this study, data were collected from 424 patients, exceeding the recommended minimum and ensuring robust results for both exploratory and confirmatory factor analyses. Therefore, a formal statistical power analysis was not performed, as the sample size is in line with psychometric best practices [26,27]. This sample size also aligns with the recommendations in the recent methodological literature for ensuring construct validity and reliability in scale development studies. In addition, using a larger sample (n = 424) enabled us to divide the dataset into two independent subsamples to conduct separate exploratory and confirmatory factor analyses. This approach strengthened the structural validity assessment and minimized overfitting. The large sample also improved the precision of factor loading estimates and the stability of reliability coefficients, thereby enhancing the overall psychometric quality of the scale.

### 2.5. Data Collection

A data collection instrument consisting of two parts was used in this research. The first part included an introductory information form consisting of 10 questions, and the second part included the “Patient Participation in Patient Safety Practices Scale” (PPPSPS).

The item pool study, which is the first phase in scale development studies, was conducted for the draft PPPSPS included in the second part. The draft scale consisted of 34 items from the literature [9,28,29,30,31,32,33]. The item pool was subjected to expert opinion to assess the content validity of the items, and it was finalized with 32 items.

**Figure 1:**
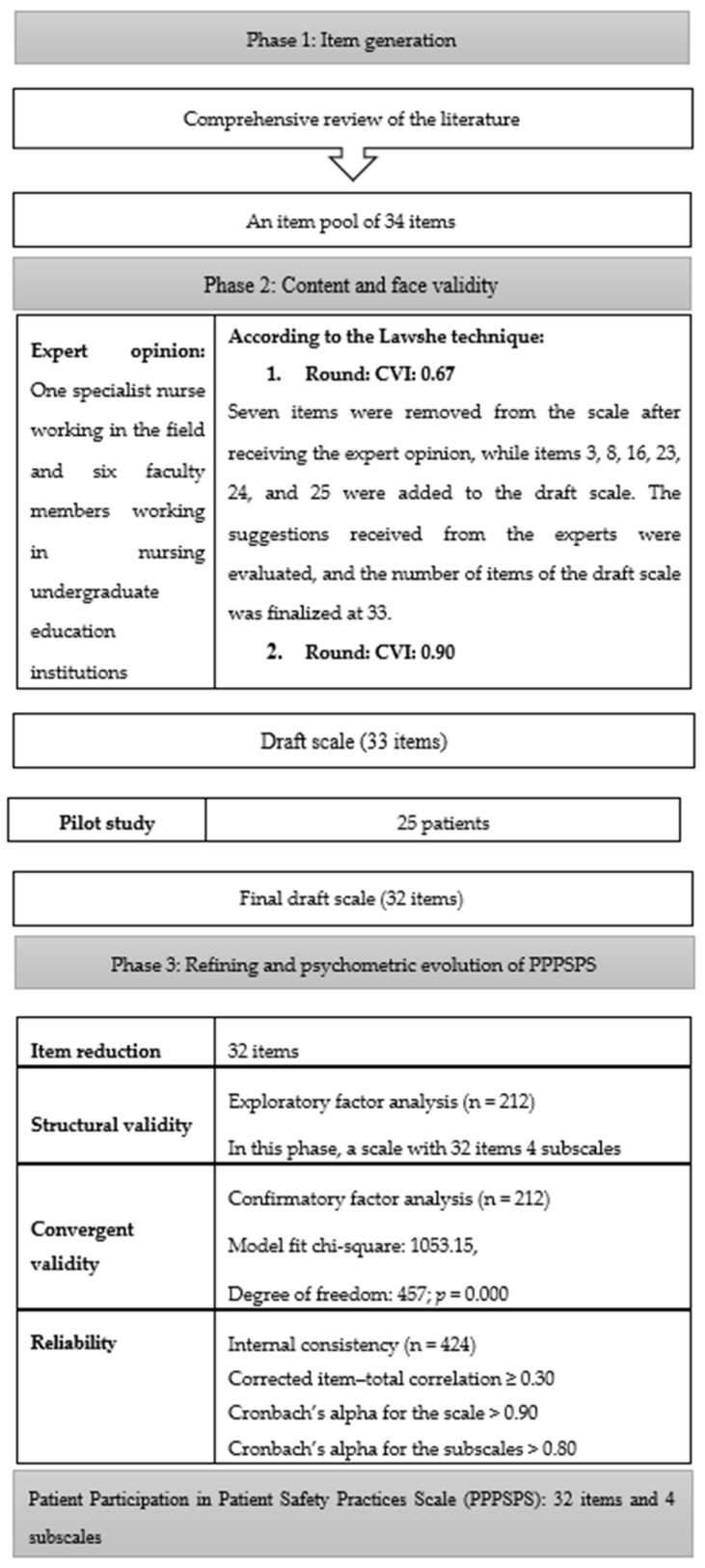
Process of developing the Patient Participation in Patient Safety Practices Scale (PPPSPS).

The PPPSPS, developed by researchers, is a scale consisting of 32 items (statements) and 4 subscales (general, infection, falls, and drugs) to determine the level of participation in patient safety practices in the hospital environment and provides measurements on the scale such as “Disagree-1”, “Agree-2”, and “Strongly Agree-3”. The total score of the scale ranges from 32 to 96. Higher scores indicate a higher level of patient participation. The development process of the Patient Participation in Patient Safety Practices Scale is shown in Figure 1.

The scale data were collected through the distribution of a questionnaire to patients. The items were filled in through reading by researchers for those patients with low sociocultural levels who struggled with reading. The scale took 10 to 15 min to fill out. To reduce potential social desirability bias during assistance, researchers were trained to read items in a neutral tone without expressing approval or disapproval. Participants were informed that their responses would remain confidential and would not affect their care.

### 2.6. Data Analysis

The data were analyzed using IBM SPSS Statistics for Windows, version 26.0 (IBM Corp., Armonk, NY, USA), [34] and LISREL 8.8 [35]. Descriptive statistics (mean, standard deviation, frequency, and percentage) were used to summarize participants’ sociodemographic characteristics and item responses.

To examine the construct validity of the scale, exploratory factor analysis (EFA) was performed on half of the dataset (n = 212) using principal component analysis with Varimax rotation. The Kaiser–Meyer–Olkin (KMO) measure of sampling adequacy and Bartlett’s test of sphericity were used to assess the suitability of the data for factor analysis.

Confirmatory factor analysis (CFA) was conducted on the second half of the data (n = 212) using LISREL 8.8 to confirm the factor structure identified through EFA. Model fit was assessed using multiple indices, including the chi-square to degrees of freedom ratio (χ²/df), Root Mean Square Error of Approximation (RMSEA), Comparative Fit Index (CFI), Goodness-of-Fit Index (GFI), Adjusted Goodness-of-Fit Index (AGFI), and Standardized Root Mean Square Residual (SRMR).

Internal consistency reliability was evaluated using Cronbach’s alpha coefficients and corrected item–total correlations, conducted in SPSS. The content validity of the scale was assessed using Lawshe’s content validity ratio (CVR) and content validity index (CVI). These calculations were performed manually based on ratings from a panel of subject matter experts [36].

### 2.7. Ethical Considerations

Non-Interventional Research Ethics Committee approval (Decision date: 21 May 2021, Decision no: 2021/15-17) and permission were received from the training and research hospital where the research was conducted and the Provincial Directorate of Health (2021/39). The study adhered to the ethical principles outlined in the Helsinki Declaration and the Personal Data Protection Act. The participants were provided with detailed information about the aim, subject, and scope of the study on the first page of the questionnaire. In addition, verbal and written informed consent were obtained, including regarding voluntary participation, duration of the questionnaire, and anonymity of the responses; thus, patients who agreed were allowed to complete the questionnaire. The participants received no payment.

## 3. Results

In this section, the characteristics of the patients who participated in the study and the stages of development of the PPPSPS are discussed.

### 3.1. Participant Characteristics

A total of 36.3% of the patients included in the study were aged 70 years or older. The average age of the participants was 61.94 ± 17.49 years, and 50.7% were male. In addition, 88.0% of the patients were literate, 46.9% were primary school graduates, and 40.1% were retired, while the income status of 47.6% of the participants was equal to expenses, 55.0% were living in a metropolitan city, 55.4% were admitted for surgical services, and the duration of hospitalization of 76.9% was 2–5 days (cited in Supplementary Table S1).

### 3.2. Phases of the Patient Participation in Patient Safety Practices Scale Development

#### 3.2.1. Creation of the Item Pool

A literature review was conducted first to develop the PPPSPS [10,29,30,31,32,33,34]. The patient participation instruments included in the national and international literature were examined. According to the information obtained by the end of the examinations, a draft scale with a 34-item pool and a 3-point Likert scale was created.

#### 3.2.2. Expert Opinions for Content Validity

The extent to which each item serves a purpose is determined via content validity. Therefore, it is necessary to include statements with high power to represent the subject on a scale instead of unrelated statements external to the subject being studied [37].

The PPPSPS was examined in terms of structure and content by a nurse working in the field and a total of seven experts selected from six faculty members working in nursing undergraduate education institutions who had previously performed a scale development study. In accordance with the Lawshe technique, the content validity index was determined to be 0.67 on the basis of the opinions expressed by seven experts regarding the items. For the draft scale, items 4, 20, 21, 22, 23, 27, and 34 were removed from the scale after expert opinions. Items 3, 8, 16, 23, 24, and 25, containing important subscales of issues related to patient safety, were added to the draft scale based on the suggestions of experts. The scale was finalized with 33 items. The corrected version of the scale was submitted to the experts, and the content validity index (CVI) of the draft scale was determined to be 0.90.

#### 3.2.3. Pilot Application of the Draft Scale

After the expert opinion, a pilot application was carried out to obtain the opinions of all participants about the comprehensibility of the items. The pilot application can be carried out with 10–15 participants. The participant group of the pilot application is associated with the variable to be measured and the target group. Characteristics such as the age, educational level, and gender of the group in the sample of the pilot application should be the same as those of the target group of the original scale [38]. The scale, which was finalized after the expert opinion, was applied to 25 patients with similar characteristics to those included in the research as part of the pilot application. In the study, the people who were included in the pilot application were not included in the sampling. Opinions were obtained from all participants about the comprehensibility of the items. In the pilot application, the statement “I am allowed to participate in decisions related to patient safety practices” was removed from the draft scale since it was not found to be comprehensible by the patients, and no corrections were made to other items. After this phase, the number of items was reduced to 32.

In the data analysis phase of the scale, the data were divided into two equal parts within the scope of structural validity studies; exploratory factor analysis was performed with the first group of data, and confirmatory factor analysis was performed with the second group of data.

### 3.3. Exploratory Factor Analysis (EFA)

To determine the validity of the factorial structure of the PPPSPS, basic component factor analysis was performed on 212 units of data. The values of KMO = 0.850 (Kaiser–Meyer–Olkin measure of sampling adequacy) and (Bartlett’s test of sphericity) chi-square = 7490.363 (*p* < 0.001), obtained according to the analysis results performed with the Varimax vertical rotation method, revealed that the data matrix had a factorizable structure. As a result of the analysis, six factors with eigenvalues above 1 were found. All items in the scale were collected in four subscales; however, the fifth and sixth factors of the nine items were randomly distributed. Therefore, the analysis was repeated by defining the number of factors as four, and the analysis results were reported.

These four factors explained 70.613% of the total variance in the data matrix. The eigenvalue of the first factor was 12.381, and the percentage of variance explained was 38.689. The eigenvalue of the second factor was 4.425, and the variance explanation percentage was 13.829. The eigenvalue of the third factor was 3.351, and the variance explanation percentage was 10.471. The eigenvalue of the fourth factor was 2.440, and the variance explanation percentage was 7.624 (cited in Supplementary Table S1).

Factor loadings represent the degree of association between each item and the underlying latent factor and are interpreted similarly to correlation coefficients. Higher factor loadings indicate a stronger relationship with the factor.

There were 11 items under the first factor, general, and these items had factor loads varying between 0.485 and 0.838. The second factor, infection, consisted of 10 items. The factor loads of the items in this factor varied between 0.542 and 0.791. The third factor, falls, consisted of six items. The factor loads of the items in this factor were in the range of 0.497–0.787. The remaining five items on the scale were included in the factor drugs, and the factor loads of these items varied between 0.471 and 0.791. The factor loads and items of the factors obtained as a result of exploratory factor analysis are given in Table 1.

### 3.4. Confirmatory Factor Analysis (CFA)

Taking into account the four-factor structure obtained as a result of exploratory factor analysis with the other half of the data obtained by the preliminary application (n = 212), a model consisting of 32 items and 4 factors was defined, and this hypothetical model was tested to determine whether the model had data compatibility by conducting confirmatory factor analysis. As a result of the analysis, the model fit chi-square value was 1053.15, with degrees of freedom of 457 and *p* = 0.000. Since the chi-square value was significant, other goodness-of-fit indices were examined for model data compliance. The results of the analysis revealed that GFI = 0.930, AGFI = 0.920, CFI = 1.000, RMSEA = 0.079, and SRMR = 0.079. The chi-square/sd ratio was 2.3. When the goodness-of-fit indices and error indices of the model data are considered together, the model data fit of the tested data is quite high at 25. These findings support that the PPPSPS has a four-dimensional structure. The factor loads of the factors obtained as a result of the confirmatory factor analysis are given in Supplementary Table S2.

As shown in Table S2, according to the results of the confirmatory factor analysis, the factor loads of the items varied between 0.27 and 0.67 for the first factor (item validity coefficients), between 0.37 and 0.75 for the second factor, between 0.59 and 0.72 for the third factor, and between 0.44 and 0.63 for the fourth factor. The R2 (item reliability) values of the items varied between 0.08 and 0.45 for the first factor, between 0.14 and 0.57 for the second factor, between 0.19 and 0.52 for the third factor, and between 0.19 and 0.40 for the fourth factor. All estimated parameters were found to be significant at a significance level of *p* ≤ 0.01. The T statistics for the factor loads are given in the last column of the table. The T values indicated that the estimated factor loads were significant at the significance level of *p* ≤ 0.01. The item-level explained variances (R² values) from the confirmatory factor analysis ranged from 0.08 to 0.57, as presented in Supplementary Table S2. Accordingly, item uniqueness (i.e., error variance) ranged from 0.43 to 0.92, indicating a mix of shared and unique variance across items. These values fall within acceptable limits and suggest that the items vary in the extent to which they are influenced by the latent construct.

**Figure 2:**
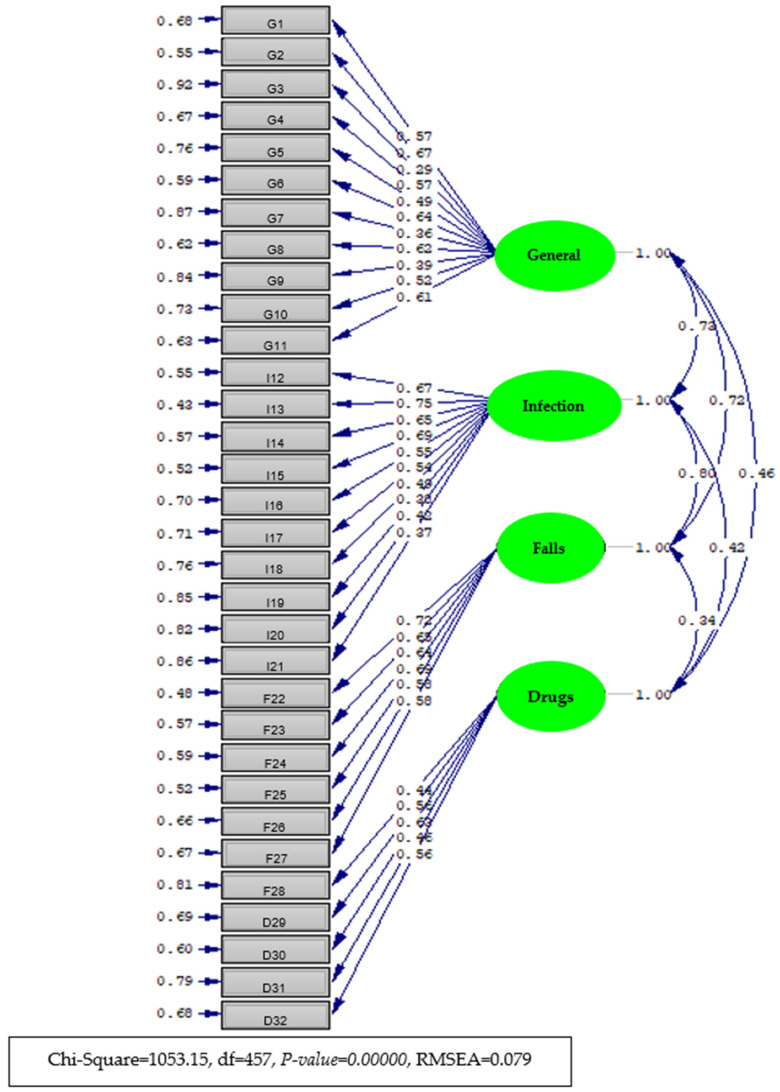
Diagram of the item-factor loads (with standard coefficients) estimated by confirmatory factor analysis of the Patient Participation in Patient Safety Practices Scale.

The path diagram of the CFA results is given in Diagram 1. Standardized factor loads (with estimated error coefficients in parentheses) and correlation coefficients between the subscales are also shown in the diagram (Figure 2).

### 3.5. Findings of the Internal Consistency Reliability Analysis

The entire dataset of the pretrial application (n = 424) was used for internal consistency reliability analysis of the overall PPPSPS and its subscales.

The internal consistency reliability coefficients of the overall PPPSPS and its subscales were obtained by determining Cronbach’s alpha reliability and are given in Table 2 below.

The Cronbach’s alpha internal consistency reliability coefficient of the general subscale was α = 0.899. The Cronbach’s alpha internal consistency reliability coefficient of the infection subscale was α = 0.932. The Cronbach’s alpha internal consistency reliability coefficient of the falls subscale was α = 0.806, whereas it was α = 0.799 for the drugs subscale. The Cronbach’s alpha internal consistency reliability coefficient for the overall PPPSPS was found to be α = 0.922. Items in the relevant subscales contributed to a high increase in reliability. As shown in Table 2, removing any item from the scale did not increase the reliability of the scale.

As shown in Supplementary Table S3, the correlations between the overall scale and its subscales and between the subscales were highly statistically significant. According to the results of the validity and reliability analyses of the pretrial PPPSPS, the scale was a valid and reliable measurement instrument.

## 4. Discussion

In this study, a 32-item PPPSPS designed to evaluate patient participation in patient safety practices was developed, and its psychometric properties were examined. In the literature, patient participation is considered the participation of patients in nursing care in certain disease groups [15,17,28]. However, the direct participation of patients in all practices is essential for ensuring patient safety. Accordingly, it is necessary to evaluate patients’ participation in the process and develop appropriate strategies. In this study, the Patient Participation in Patient Safety Practices Scale, which can be applied in all patient groups, was developed to obtain data that would meet this requirement. To the best of our knowledge, this study is the first in Turkey and in the international literature. The instrument was cognitively tested on the target population, and the adapted version was tested on a larger sample.

It is necessary to obtain an expert opinion on the item pool, which is the first of the scale development phases, in terms of content validity. The content validity indicates that each item in the scale serves the entire scale and its purpose [37]. In this study, opinions were obtained from seven experts for the content validity of the Patient Participation in Patient Safety Practices Scale, and the Lawshe technique was used to evaluate the expert opinions [37,39]. For content validity, it is recommended that the number of experts should be at least 3 people and at most 20 people and particularly be an odd number [40]. Therefore, the content validity index of the seven expert opinions was 0.896. Yurdugul’s [41] approach was considered. A CVI value of 0.80 was suggested as a criterion, and it was decided that there was a consensus among the experts. The level of consensus among the experts was high, and each item on the scale served the entirety and purpose of the scale. Item-level content validity ratio (CVR) values were calculated using Lawshe’s method based on the ratings of seven experts. These values are presented in Supplementary Table S4.

The PPPSPS consists of four factors: general, infection, falls, and drugs. Previous studies have shown that patients accept the importance of participation and their roles in their own healthcare in the infection subscale [13]. In general, regarding the infection subscale, broad support for the creation of a patient-centered care culture, which recognizes the importance of the role of patients and places patients at the center of the patient safety movement, can encourage a change toward the hand hygiene paradigm in relation to hand washing, which is highly important in preventing infections [8]. In terms of the falls subscale, bedside shift reports with patient participation can reduce patient falls, and the information is more objective when the patient is involved, which enables patients to be included more in their own healing process [42]. The Joint Commission established the “Speak Up” program for patient safety in March 2002. This program aims to help patients and their relatives take an active role in patient care. The patient is considered an informed member of the medical team, and they are encouraged to talk to prevent medical errors, giving simple advice on their actions. The “Speak Up” program provides various brochures and videos for patients with instructions on their participation in the prevention of risks that may threaten patient safety, such as medication errors or hospital infections [43]. The structural validity of the scale was assessed by conducting EFA and then CFA. In addition, the CFA results revealed the acceptable fit of the model to the observed data [44].

The scale’s four subscales each reflect critical domains of patient involvement in safety. The general subscale represents the patient’s overall awareness and perceived responsibility in ensuring their safety, echoing the WHO’s emphasis on patients as partners in care [45]. The infection subscale aligns with global standards in hand hygiene and infection prevention and reflects the role of patients in influencing hygiene compliance [8]. The falls subscale incorporates evidence-based strategies such as proactive behavior and environmental awareness to prevent inpatient falls, which are among the most common safety events [42]. Finally, the drugs subscale emphasizes communication, transparency, and patient vigilance regarding medications—elements frequently cited in strategies to reduce medication errors [6]. Together, these subscales provide a multidimensional framework for understanding patient roles in real-time clinical safety activities.

Although previous instruments such as the Patient Activation Measure [16], the Patient Health Engagement Scale [15], and the Patient Involvement Index [10] have contributed to understanding general engagement and activation levels, these tools do not focus on safety-specific practices during hospitalization. The PPPSPS differs in that it assesses direct patient behaviors that contribute to preventing adverse events, such as medication verification, fall prevention actions, and infection control behaviors. Unlike existing tools, the PPPSPS was developed specifically for use in clinical safety contexts and reflects behaviors relevant to the acute care environment.

While all four subscales of the PPPSPS demonstrated acceptable to excellent internal consistency, the general and infection subscales had Cronbach’s alpha values of 0.799, which are slightly below the commonly recommended threshold of 0.80 for established scales [46,47]. Although these values are statistically acceptable, they suggest the potential for refinement to enhance measurement precision. In addition, several items (e.g., items 3, 7, 9, 19, and 21) exhibited factor loadings below the conventional 0.40 threshold in the confirmatory factor analysis. These items were retained based on their conceptual significance and theoretical alignment with the multidimensional nature of patient participation. For instance, item 3 emphasizes communication and mutual understanding, which is foundational to participation but may vary in salience across patient populations. Item 7 represents autonomy in decision-making, while item 9 reflects access to safety-related information—both of which are essential but sometimes contextually limited in practice. Items 19 and 21 pertain to proactive hygiene-related behaviors, which can be infrequent yet are crucial for comprehensive safety involvement. Despite their lower loadings, the inclusion of these items did not compromise the model fit or internal consistency, and they enrich the instrument’s content validity by capturing less frequently measured, but theoretically important, aspects of participation.

One possible explanation for these slightly lower reliability coefficients may be the limited number of items in these subscales or the broad conceptual range of the constructs they attempt to capture. According to [26], scales with fewer items are more susceptible to lower internal consistency, especially when the construct is multidimensional or abstract in nature. To improve reliability, future research may consider revising or expanding item content to ensure conceptual clarity and alignment. This could include rewording items for cultural relevance, adding more behaviorally specific indicators, or conducting qualitative item evaluations such as cognitive interviews to identify ambiguities in item interpretation. Additionally, testing the scale in different clinical settings or patient populations may help establish stronger generalizability and psychometric robustness.

While the PPPSPS was designed for broad applicability across patient populations, the present validation study focused on adult inpatients. This population was selected because hospitalized patients are more likely to observe and experience safety-related practices directly, making them particularly suited to evaluating participation behaviors during care. Nevertheless, further research is needed to test the scale’s applicability in outpatient, pediatric, and cross-cultural settings to strengthen its external validity.

The PPPSPS can be utilized not only as a research tool but also as a practical instrument within hospital-based quality improvement initiatives. By identifying areas where patient participation in safety practices is limited, the scale can help guide targeted interventions, such as patient education programs or staff training, to support shared responsibility. Furthermore, the scale may contribute to institutional self-assessment processes within national and international accreditation frameworks, such as Turkey’s Health Quality Standards (SKSs), the Joint Commission’s Speak Up initiatives, or Magnet Recognition Program standards, which emphasize patient engagement as a core dimension of care quality. Routine administration of the PPPSPS can serve as a monitoring tool to track improvements in patient involvement over time and ensure alignment with evolving patient safety goals.

## 5. Limitations

There are several limitations to this research. First, the use of a convenience sample may affect the generalizability of the findings, potentially resulting in the underrepresentation or overrepresentation of certain patient groups. The results are also limited to the self-reported responses of the patients, which may be subject to recall or response bias.

Additionally, data were collected through interviewer-administered questionnaires, and assistance was provided to some participants with low literacy. Since all data were collected by a single researcher, efforts were made to minimize social desirability bias by maintaining a neutral tone, using a standardized data collection script, and assuring participants of the confidentiality and anonymity of their responses. Nevertheless, the risk of social desirability bias remains a potential limitation.

## 6. Conclusions

The results of the analysis of the Patient Participation in Patient Safety Practices Scale (PPPSPS) support its internal structural validity. Consequently, the contribution of the items to the reliability of the test was adequate, and the items of the scale were consistent in measuring the desired properties. The Turkish version of the PPPSPS, consisting of 32 items and 4 subscales—namely, general (11 items), infection (10 items), falls (6 items), and drugs (5 items)—is a valid and reliable instrument for evaluating patient participation in the Turkish population.

In addition to its psychometric strength, the PPPSPS provides a practical framework for monitoring and promoting patient involvement in safety-related behaviors in clinical practice. It can be utilized in hospital-based quality improvement programs to identify weak points in patient engagement and design targeted interventions. Moreover, the tool offers valuable insights for nurse leaders, enabling them to promote participatory care models and integrate patient feedback into safety strategies, thereby strengthening both nursing leadership practices and a proactive safety culture across care teams.

For broader impact, future research should focus on evaluating the scale’s measurement invariance across different cultural and clinical contexts, as well as its sensitivity to changes over time through longitudinal studies. Investigating how PPPSPS scores correlate with actual patient outcomes and how it can be integrated into staff training, patient education, and accreditation processes will further support its applicability in national and international healthcare settings.

## Figures and Tables

**Table 1:** The item-factor loads of the factors obtained as a result of exploratory factor analysis.

	FACTORS/ITEMS	Factor Load
	General	
1.	It is important that I also participate and be willing to ensure my safety in the hospital environment (not to be harmed by the hospital environment).	0.772
2.	I can prevent medical errors when I give warnings by noticing errors in the hospital environment.	0.638
3.	Good communication and listening to patients are important for them to participate in their own care.	0.679
4.	It is important that health professionals support me to participate in my own care.	0.475
5.	It is important to provide training to healthcare professionals to prevent negative reactions to my warnings about patient safety practices.	0.618
6.	It is ensured that I learn the necessary information about patient safety practices (such as preventing falls, preventing infection).	0.660
7.	I can easily ask questions about patient safety.	0.756
8.	I can access materials such as informative booklets, brochures, etc., on patient safety.	0.550
9.	If I think I have been mistaken for another patient, I will share this with a healthcare professional.	0.838
10.	I also participate in all kinds of care practices that will be performed on me (for instance, hand–face hygiene, foot care).	0.485
11.	When I am discharged, I think that, thanks to my participation, I will be able to properly perform my home care (care practices, correctly taking my drugs).	0.684
	Infection	
12.	I wash my hands as the most effective method to avoid getting infections.	0.791
13.	I wash my hands before touching medical equipment and injured areas.	0.744
14.	I wash my hands before meals.	0.742
15.	I wash my hands after meals.	0.628
16.	I wash my hands before using the toilet.	0.692
17.	I wash my hands after using the toilet.	0.553
18.	I wash my hands before leaving my room.	0.710
19.	I wash my hands before entering my room.	0.542
20.	I use alcohol-based antiseptic solution or cologne when I cannot wash my hands (if the sink is dirty, there is no soap, towels, etc.).	0.670
21.	I make sure that relatives of patients and visitors wash their hands.	0.774
	Falls	
22.	It is important that my recommendations for preventing falls are considered by healthcare professionals.	0.787
23.	I prefer nonslip clogs at the hospital.	0.767
24.	I definitely turn on the lights in places with poor lighting.	0.587
25.	I stand up in a controlled manner.	0.703
26.	If I feel dizzy or weak, I do not get up on my own and ask for help.	0.610
27.	I need nurses to give warnings and reminders about fall prevention.	0.497
	Drugs	
28.	I make sure that the administered drugs are assigned to me.	0.498
29.	I receive information from doctors/nurses about the reasons for using the drugs given to me.	0.673
30.	During my treatment, I ask healthcare professionals questions about their use (how to take them) and the duration of usage in drugs administration.	0.586
31.	I talk to health professionals about all the possible allergies or side effects of the administered drugs.	0.791
32.	If I have brought the drugs I take at home with me during my stay in the hospital, I will inform my nurse.	0.471

**Table 2:** Cronbach’s alpha of the Patient Participation in Patient Safety Practices Scale.

FACTORS/ITEMS	The Average of the Subscale If the Item Was Removed	The Variance of the Subscale If the Item Was Removed	Correlation of the Item with the Subscale	Reliability of the Subscale If the Item Was Removed
1	20.97	18.399	0.708	0.875
2	20.99	18.057	0.640	0.878
3	20.73	18.368	0.611	0.879
4	20.90	18.184	0.714	0.874
5	20.86	18.279	0.620	0.879
6	21.21	18.015	0.614	0.879
7	21.04	18.327	0.589	0.881
8	21.65	19.552	0.352	0.895
9	20.63	18.166	0.643	0.878
10	21.03	18.155	0.650	0.877
11	21.03	18.134	0.611	0.880
Overall	23.10	21.937	-	-
General Subscale Cronbach’s Alpha =	0.899			
12	0.875	16.437	0.689	0.883
13	0.878	15.852	0.750	0.878
14	0.879	15.941	0.776	0.877
15	0.874	15.871	0.787	0.877
16	0.879	16.047	0.536	0.894
17	0.879	16.255	0.733	0.880
18	0.881	15.793	0.544	0.895
19	0.895	16.008	0.593	0.889
20	0.878	16.558	0.613	0.887
21	0.877	16.289	0.539	0.893
Overall	23.65	19.655	-	-
Infection Subscale Cronbach’s Alpha =	0.896			
22	11.40	5.900	0.531	0.784
23	11.47	5.635	0.520	0.785
24	11.50	5.206	0.639	0.758
25	11.48	5.144	0.673	0.750
26	11.38	5.575	0.506	0.789
27	11.73	5.220	0.533	0.785
Overall	13.79	7.550	-	-
Falls Subscale Cronbach’s Alpha =	0.806			
28	8.20	4.425	0.551	0.771
29	8.26	4.184	0.699	0.724
30	8.25	4.359	0.639	0.744
31	8.56	4.205	0.617	0.750
32	7.96	4.916	0.414	0.810
Overall	10.31	6.573	-	-
Drugs Subscale Cronbach’s Alpha =	0.799			
Overall Cronbach’s Alpha =	0.922			

## Data Availability

The raw data supporting the conclusions of this article will be made available by the authors on request.

## Supplementary Materials

The following supporting information can be downloaded at: https://www.mdpi.com/article/10.3390/healthcare13121387/s1, Table S1. The results of the exploratory factor analysis of the Patient Participation in Patient Safety Practices Scale; Table S2. Factor loads obtained by confirmatory factor analysis of the Patient Participation in Patient Safety Practices Scale. Explained variance (R2) and T statistics; Table S3. The correlation coefficients between all the subscales of the Patient Participation in Patient Safety Practices Scale; Table S4. Item-level CVR values for the Patient Participation in Patient Safety Practices Scale (PPPSPS) based on expert evaluations.

## Abbreviations

The following abbreviations are used in this manuscript:

PPPSPS	Patient Participation in Patient Safety Practices Scale
CVR	Content Validity Ratio
CVI	Content Validity Index
KMO	Kaiser–Meyer–Olkin
EFA	Exploratory Factor Analysis
CFA	Confirmatory Factor Analysis
