# Supplementary material for: Patient Participation in Patient Safety Practices Scale: Development and Psychometric Evaluation of a Scale
Source: Healthcare (Basel). 2025 Jun 11;13(12):1387. doi: 10.3390/healthcare13121387 (PMC12192807; doi:10.3390/healthcare13121387)
Supplement: Supplementary file 1 [file healthcare-13-01387-s001.zip › healthcare-3648217-supplementary.pdf]

Table S1. The Results of the Exploratory Factor Analysis of Patient Participation in Patient Safety Practices Scale

Components/Factors	Eigenvalues	Variance Explanation Percentage
**General**	12.381	38.689
**Infection**	4.425	13.829
**Falls**	3.351	10.471
**Drugs**	2.440	7.624

Factor Inference Method: Basic Components

Table S2. Factor Loads Obtained by Confirmatory Factor Analysis of Patient Participation in Patient Safety Practices Scale. Explained Variance (R^2) and T Statistics

FACTORS/ ITEMS	Factor Load	R^2	t
**General**			
**1**	0.57	0.32	**5.98****
**2**	0.67	0.45	**8.47****
**3**	0.29	0.08	**3.20****
**4**	0.57	0.33	6.45**
**5**	0.49	0.24	6.32**
**6**	0.64	0.41	9.39**
**7**	0.36	0.13	5.10**
**8**	0.62	0.38	5.90**
**9**	0.39	0.16	4.27**
**10**	0.52	0.27	7.07**
**11**	0.61	0.37	8.43**
**Infection**			
**12**	0.67	0.45	7.66**
**13**	0.75	0.57	9.31**
**14**	0.65	0.43	7.51**
**15**	0.69	0.48	8.03**
**16**	0.55	0.30	8.76**
**17**	0.54	0.29	6.23**
**18**	0.48	0.24	7.41**
**19**	0.38	0.15	5.74**
**20**	0.42	0.18	5.42**
**21**	0.37	0.14	5.57**
**Falls**			
**22**	0.72	0.52	7.99**
**23**	0.65	0.43	9.21**
**24**	0.64	0.48	7.99**
**25**	0.69	0.34	8.87**
**26**	0.58	0.19	9.02**
**27**	0.58	0.33	8.26**
**Drugs**			
**28**	0.44	0.19	4.03**
**29**	0.56	0.31	4.59**
**30**	0.63	0.40	5.00**
**31**	0.46	0.21	3.88**
**32**	0.56	0.31	4.97**

**Significant at a level of $p \leq 0.01$

Table S3. The Correlation Coefficients Between All the Subscales of the Patient Participation in Patient Safety Practices Scale

Subscales	General	Infection	Falls	Drugs
**Infection**	0.453**	-	-	
**Falls**	0.504**	0.547**	-	
**Drugs**	0.305**	0.383**	0.296**	-
**Overall Scale**	0.799**	0.818**	0.756**	0.590**

(**) Significant at the level of $p<0.001$

Table S4. Item-level CVR Values for the Patient Participation in Patient Safety Practices Scale (PPPSPS) Based on Expert Evaluations (N = 7).

Item No	Subscale	CVR Value
G1	General	0.714
G2	General	0.714
G3	General	1
G4	General	0.429
G5	General	0.714
G6	General	1
G7	General	1
G8	General	1
G9	General	1
G10	General	1
G11	General	0.714
G12	General	0.429
I1	Infection	0.714
I2	Infection	1
I3	Infection	1
I4	Infection	1
I5	Infection	1
I6	Infection	1
I7	Infection	1
I8	Infection	1
I9	Infection	1
I10	Infection	1
F1	Falls	1
F2	Falls	1
F3	Falls	1
F4	Falls	1
F5	Falls	0.714
F6	Falls	1
M1	Drugs	0.714
M2	Drugs	1
M3	Drugs	0.714
M4	Drugs	1
M5	Drugs	1
